# Effectiveness of a Novel Mono‐Block Splint Versus a Twin‐Block Splint for Anterior Disc Displacement With Reduction: A Randomised Controlled Trial

**DOI:** 10.1111/joor.70179

**Published:** 2026-03-07

**Authors:** Zhilei Liu, Antong Wu, Wenyi Cai, Jiaqian Fan, Yunyi Yuan, Yufu Lin, Rong Zhang, Qingbin Zhang, Wei Cao

**Affiliations:** ^1^ Department of Temporomandibular Joint School and Hospital of Stomatology Guangzhou China; ^2^ Guangdong Engineering Research Center of Oral Restoration and Reconstruction, Guangzhou Key Laboratory of Basic and Applied Research of Oral Regenerative Medicine Guangzhou Medical University Guangzhou China; ^3^ Laboratory for Myology, Department of Human Movement Sciences, Faculty of Behavioural and Movement Sciences Vrije Universiteit Amsterdam, Amsterdam Movement Science Amsterdam the Netherlands; ^4^ Department of Stomatology, The Second Affiliated Hospital of Guangzhou Medical University Guangzhou Medical University Guangzhou China; ^5^ Division of Dentistry, School of Medical Sciences, Faculty of Biology, Medicine and Health Henry Royce Institute, The University of Manchester Manchester UK; ^6^ Orthodontics and Pediatric Dentistry Center School and Hospital of Stomatology Guangzhou China

**Keywords:** anterior disc displacement with reduction, anterior repositioning splint, CBCT, temporomandibular joint disorders

## Abstract

**Background:**

Anterior disc displacement with reduction (ADDwR) is a common subtype of temporomandibular disorders (TMD). Anterior repositioning splint (ARS) serves as a key conservative treatment modality. This study aimed to compare the clinical efficacy of a novel Mono‐Block splint (nMB) with that of a Twin‐Block (TB) splint in the management of ADDwR.

**Methods:**

A total of 118 patients with ADDwR were randomised to receive either an nMB or TB splint. Clinical outcomes were assessed at baseline (T0), 1 month (T1), 3 months (T3) and 6 months (T6). CBCT scans were obtained at T0 and T6, and patient satisfaction was evaluated at T6.

**Results:**

Joint noise: The nMB group showed significantly greater improvement than the TB group at T1 and T6. Joint pain: Greater symptom relief was observed in the nMB group at the third time point (T3). MMO: The nMB splint induced a significantly greater increase in MMO only at T1. The condyle–fossa relationship: CBCT evaluation revealed comparable outcomes between the two groups, with no significant difference. Patient satisfaction: The nMB splint achieved significantly higher ratings in general satisfaction, comfort and stability, while no significant differences were noted in cleaning and retention.

**Conclusion:**

The nMB splint demonstrated superior effectiveness in reducing joint noise and improving patient comfort compared to the TB splint, while both splints exhibited similar efficacy in relieving joint pain and enhancing MMO. The nMB design offers a promising, patient‐oriented approach for conservative treatment of ADDwR. Future studies will incorporate more comprehensive assessments, including MRI evaluation, and extend the follow‐up period beyond 6 months to better determine the long‐term therapeutic effectiveness of the nMB splint.

## Graphical abstract

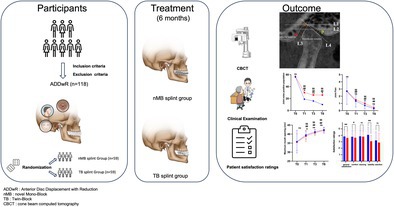

**Abbreviations**
ADDwR: Anterior disc displacement with reduction
ARS: Anterior repositioning splints
DC/TMD: Diagnostic Criteria for Temporomandibular Disorders
MB: Mono‐Block
MMO: maximum mouth opening
TB: Twin‐Block
TMD: Temporomandibular disorders
TMJ: Temporomandibular joint
*χ* ^2^ test: Chi‐square test

## 1 Introduction

Temporomandibular disorder (TMD) is among the four most prevalent conditions affecting the oral and maxillofacial region. TMD encompasses a spectrum of functional impairments involving the temporomandibular joint (TMJ), masticatory muscles and psychosocial status [1, 2]. It predominantly affects adults between the ages of 20 and 40 and occurs more frequently in females [3, 4]. Epidemiological studies estimate that approximately 10%–15% of the adult population experiences TMD symptoms [3, 5].

The primary clinical manifestations of TMD include joint noise (e.g., clicking or popping), joint pain and restricted mouth opening. Additional symptoms may involve masticatory muscle tenderness, difficulties with chewing or speaking, tinnitus, dizziness and headaches. Among the various subtypes, anterior disc displacement with reduction (ADDwR) stands out as both highly prevalent and clinically significant [6, 7].

Clinically, ADDwR is characterised by clicking sounds during mandibular movement. Typically, a loud click occurs during mouth opening, followed by a softer click upon closure, corresponding to disc recapture and subsequent displacement [8]. Magnetic resonance imaging (MRI) in these patients often shows anterior displacement of the articular disc in the closed‐mouth position, with normal disc‐condyle alignment re‐established upon opening, a key diagnostic indicator of the ‘reduction’ phenomenon [9]. In addition, cone beam computed tomography (CBCT) findings commonly reveal structural changes in patients with ADDwR, such as increased anterior joint space, decreased posterior joint space and posterior positioning of the condyle within the glenoid fossa [10, 11].

The management of ADDwR primarily emphasises conservative, non‐invasive treatment strategies, among which intraoral appliances, such as occlusal splints, play a pivotal role [12, 13]. These appliances function to redistribute occlusal forces, correct discrepancies in the spatial relationship among the occlusion, masticatory muscles, condyle and articular disc and reduce excessive muscular activity. Through these mechanisms, they aim to progressively restore a stable disc‐condyle relationship while minimising further damage to the TMJ [14]. Among the various splint types, the anterior repositioning splint (ARS) has demonstrated efficacy in treating ADDwR [13]. The ARS repositions the mandible anteriorly, thereby altering the distribution of joint space, promoting disc recapture and alleviating hallmark symptoms such as joint clicking and masticatory muscle pain [14, 15, 16].

The Twin‐Block (TB) splint can serve as a representative ARS for the management of TMD, as it actively positions the mandible downward and forward to facilitate recapture of the anteriorly displaced disc [17]. However, clinically used TB splints may fail to maintain stable mandibular advancement during sleep, especially in patients with a habitual mouth‐breathing pattern, which can compromise treatment consistency [18, 19, 20]. In contrast, Mono‐Block (MB) splints offer greater stability but are often bulky, resulting in significantly reduced comfort and patient compliance [21]. To address these issues, we developed a novel Mono‐Block (nMB) splint. Although conceptually based on existing ARS principles, the clinical efficacy of this redesigned nMB splint in managing ADDwR has not yet been validated in a large‐scale, controlled clinical trial.

Therefore, this randomised controlled trial was conducted to compare the therapeutic efficacy of the nMB splint with that of the TB splint, which served as a positive control. Patients diagnosed with ADDwR were randomly allocated to one of the two treatment groups. Treatment outcomes were assessed through multiple clinical parameters, including joint noise, maximum mouth opening (MMO), joint pain (measured via a Visual Analog Scale, VAS), CBCT analysis and patient satisfaction ratings (also measured via a VAS). This study aimed to evaluate the clinical value of the nMB splint in the management of ADDwR and to provide insights into optimising effective, patient‐oriented strategies for managing TMD and ADDwR.

## 2 Methods and Materials

### 2.1 Participants

Patients diagnosed with ADDwR at the Temporomandibular Joint Department of the Affiliated Stomatology Hospital of Guangzhou Medical University between December 2023 and December 2024 were considered for inclusion. All participants provided written informed consent prior to enrollment. The study was conducted in accordance with the Declaration of Helsinki and was approved by the Ethics Committee of the Affiliated Stomatology Hospital of Guangzhou Medical University (Ethics Approval Number: LCYJ20250618004). Inclusion criteria: (a) Diagnosis of TMD based on the Diagnostic Criteria for Temporomandibular Disorders (DC/TMD); (b) MRI confirming ADDwR; (c) Relatively complete dentition in both the maxillary and mandibular arches; and (d) Good compliance and willingness to adhere to treatment protocols and follow‐up visits. Exclusion criteria: (a) Moderately to severely limited mouth opening (maximum mouth opening < 20 mm); (b) Significant tooth loss in either dental arch compromising splint fitting or retention; (c) History of orthognathic surgery, prior TMD treatment, or maxillofacial trauma; (d) Presence of severe congenital, systemic, or hereditary conditions, or psychological disorders affecting compliance; and (e) Other exclusion factors as deemed appropriate by the investigators.

### 2.2 Randomisation and Allocation Concealment

A total of 118 participants were randomised in a 1:1 ratio to the nMB group (*n* = 59) or the TB group (*n* = 59). The randomisation sequence was computer‐generated by an independent statistician using variable block sizes of 4 and 6 to ensure balanced allocation and reduce predictability. No stratified randomisation was applied. The randomisation sequence was safeguarded by a coordinator not involved in trial implementation and stored in sequentially numbered, opaque, sealed envelopes (SNOSE). After confirming participant eligibility and completing baseline assessments, a research assistant opened the next envelope in sequence to determine group assignment. Participant enrollment was conducted by study clinicians, and intervention assignment was carried out by a research assistant. Treating clinicians and participants could not foresee the allocation. Outcome assessors and the trial statistician remained blinded to group assignments throughout follow‐up and data analysis.

### 2.3 Blinding

This study was conducted as an open‐label randomised controlled trial. Because of the apparent differences between the two splint designs, neither patients nor treating clinicians were blinded to group allocation. Clinical outcome assessments were also performed without blinding. However, the statistician responsible for data analysis was blinded to group allocation to minimise bias.

### 2.4 Splint Treatment Protocol

A total of 118 eligible patients were enrolled and randomly assigned to two groups: the nMB splint group (*n* = 59) and the TB splint group (*n* = 59). The nMB splint is a one‐piece ARS that covers the full dental arch (Figures S1A,B and S2A–C). Before fabrication, dental impressions and occlusal registrations were obtained. Patients were instructed to perform repeated mandibular opening and closing movements to facilitate proprioceptive relaxation. Once joint noise was eliminated at MMO, the mandible was gently guided into the minimal protruded position that did not induce muscular fatigue or discomfort. This position was identified as the optimal therapeutic protrusion [22]. Intraoral scans and wax bite registrations were captured in this position using a digital intraoral scanner.

All procedures were performed by the same experienced clinician to ensure standardisation. The acquired records were submitted to the laboratory, where all splints were fabricated by a single dedicated technician to minimise inter‐operator variability. For the TB splint group (Figures S1C,D and S2D–F), the same clinical and laboratory workflow was followed to ensure protocol consistency across both groups.

Patients were instructed to wear the splints only at nighttime while sleeping. The nMB splint was designed with an anterior breathing space to prevent airway obstruction during sleep. Follow‐up visits were scheduled at 1 month (T1), 3 months (T3) and 6 months (T6) after splint delivery to evaluate treatment progress, adjust the appliance if necessary, and reinforce patient compliance.

### 2.5 Clinical Evaluations

Clinical data were collected at four time points: baseline (T0), 1 month (T1), 3 months (T3) and 6 months (T6) following splint delivery. All assessments were performed by the same experienced clinician following a standardised protocol outlined in our previous research [23]. (a) Joint noise: Bilateral TMJ sounds were assessed by palpation. Patients were instructed to perform three repetitions of mandibular opening and closing, as well as anterior–posterior and lateral excursions. Any audible clicking, popping, or crepitus was recorded and classified as abnormal. (b) Joint pain: Subjective pain intensity was evaluated using a 5‐point (0–5) VAS, where 0 indicated no pain, and 5 represented the most severe pain perceived. (c) MMO: MMO was measured as the interincisal distance (in millimetres) between the upper and lower central incisors during pain‐free maximal mouth opening. All adverse events and patient complaints were recorded at each follow‐up visit.

### 2.6 CBCT Analysis

**FIGURE 1:**
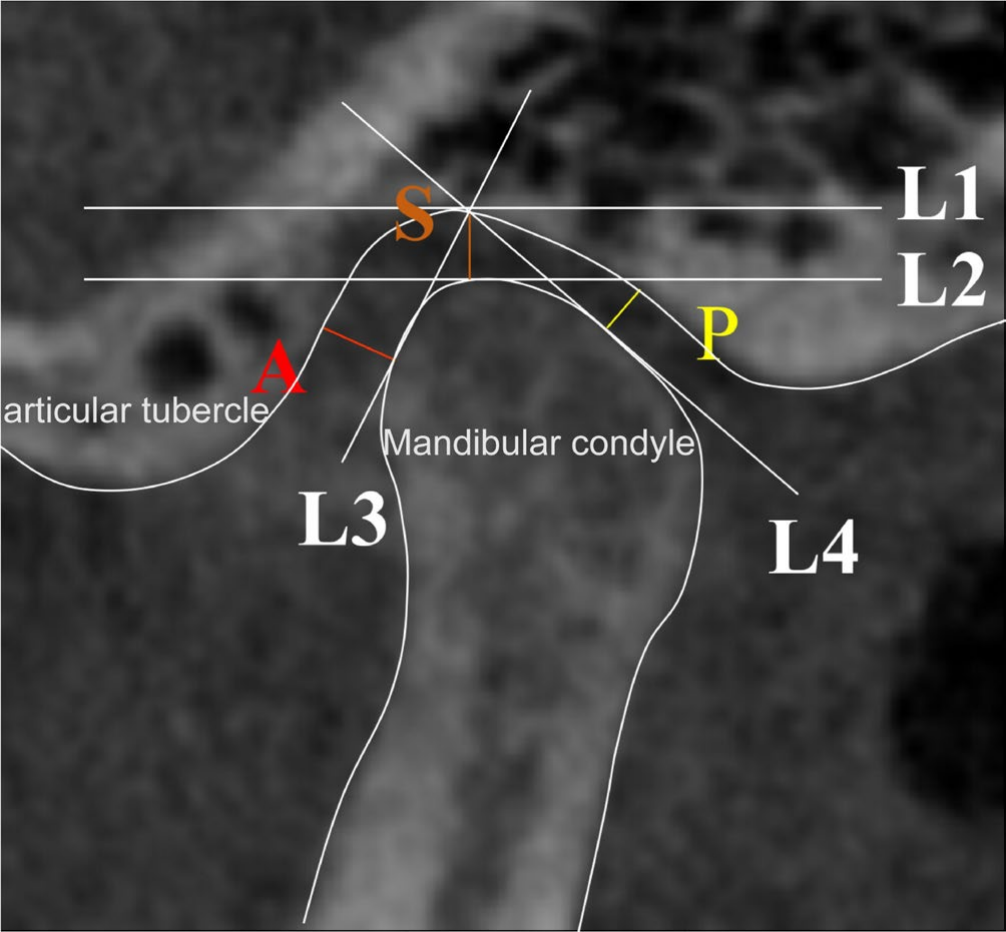
CBCT measurement based on Kamelchuk's method. L1: Horizontal line parallel to the Frankfort Horizontal (FH) plane, tangent to the roof of the glenoid fossa. L2: Horizontal line parallel to the FH plane, tangent to the superior edge of the condyle. L3: Tangent line to the anterior surface of the condyle. L4: Tangent line to the posterior surface of the condyle. A, Anterior joint space; P, Posterior joint space; S, Superior joint space.

The condyle–fossa relationship was evaluated using the method proposed by Kamelchuk [10, 24]. Specifically, two horizontal lines (L1 and L2) were drawn parallel to the Frankfort horizontal (FH) plane: L1 was tangent to the roof of the glenoid fossa, and L2 was tangent to the superior surface of the condyle. The perpendicular distance between L1 and L2 was recorded as the superior joint space (S). Tangents L3 and L4 were drawn along the anterior and posterior margins of the condyle, passing through the tangent points of the superior margin of the articular fossa. Plumb lines perpendicular to L3 and L4 indicated the anterior joint space (A) and the posterior joint space (P), respectively (Figure 1). The Pullinger method [10, 25] was used to assess condylar position in the articular fossa: A value of ln(P/A) < −0.25 indicates that the condyle is in a posterior position; ln(P/A) > 0.25 indicates that the condyle is in an anterior position; and a value of ln(P/A) between −0.25 and +0.25 indicates that the condyle is in an essentially neutral position.

### 2.7 Patient Satisfaction Ratings

All patients evaluated the comfort and usability of the occlusal splints by rating their satisfaction across five aspects: general satisfaction, ease of cleaning, stability, retention and comfort. Each parameter was assessed using a 5‐point VAS [26] based on their subjective experience, where 0 indicated complete dissatisfaction and five indicated complete satisfaction.

### 2.8 Statistical Analysis

Statistical analyses were performed using SPSS version 27.0 (IBM Corp., Armonk, NY, USA). The Shapiro–Wilk test was used to assess the normality of the quantitative data. Depending on data type and distribution, the following tests were applied to evaluate differences between the two groups at various time points: independent samples *t*‐test, Mann–Whitney *U* test, Chi‐square *χ* ^2^ test, Wilcoxon signed‐rank test, and paired *t*‐test. A *p*‐value < 0.05 was considered statistically significant.

### 2.9 Participant Flow

**FIGURE 2:**
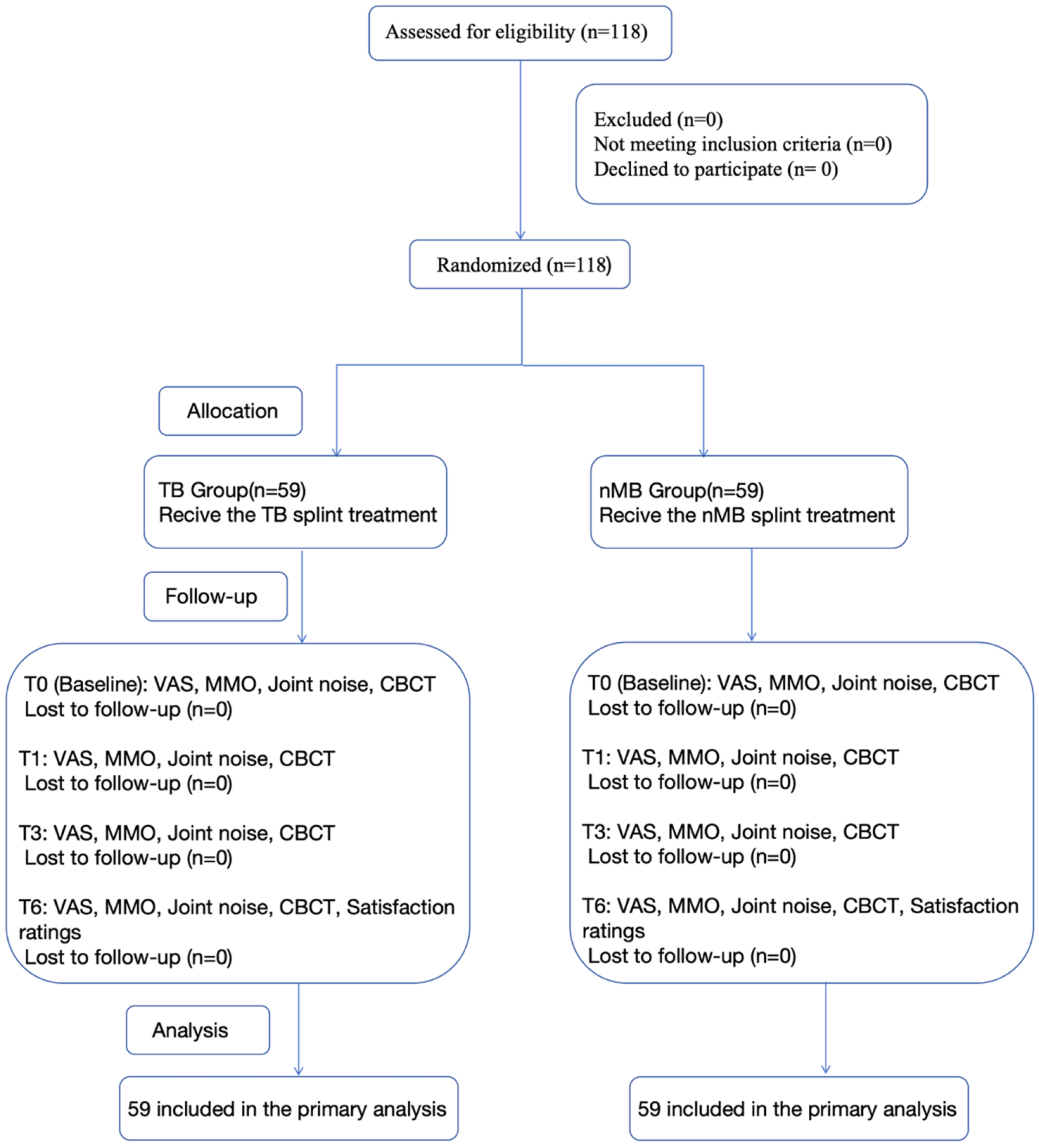
CONSORT flow diagram of participant enrollment, allocation, follow‐up and analysis. A total of 118 patients with anterior disc displacement with reduction (ADDwR) were assessed for eligibility, and all met inclusion criteria. Participants were randomised in a 1:1 ratio to either the TB splint group (*n* = 59) or the nMB splint group (*n* = 59). Both groups underwent baseline assessments (T0: VAS, MMO, joint noise, CBCT), followed by evaluations at 1 month (T1), 3 months (T3) and 6 months (T6). Patient satisfaction ratings were collected at T6. No participants were lost to follow‐up, and all 118 patients were included in the primary analysis.

A total of 118 patients diagnosed with ADDwR were screened and found eligible for inclusion. All participants were randomised in a 1:1 ratio to receive either the nMB splint or the TB splint (*n* = 59 per group). No participants were excluded, refused to participate, or lost to follow‐up at any time during the study period. All randomised participants completed the intervention and were included in the final analysis (Figure 2).

## 3 Results

### 3.1 Participant Flow and Adverse Events

All 118 randomised participants completed the study, with no loss to follow‐up or withdrawals (Figure 2). No adverse events or patient complaints were reported during the study period.

### 3.2 Basic Characteristics

**FIGURE 3:**
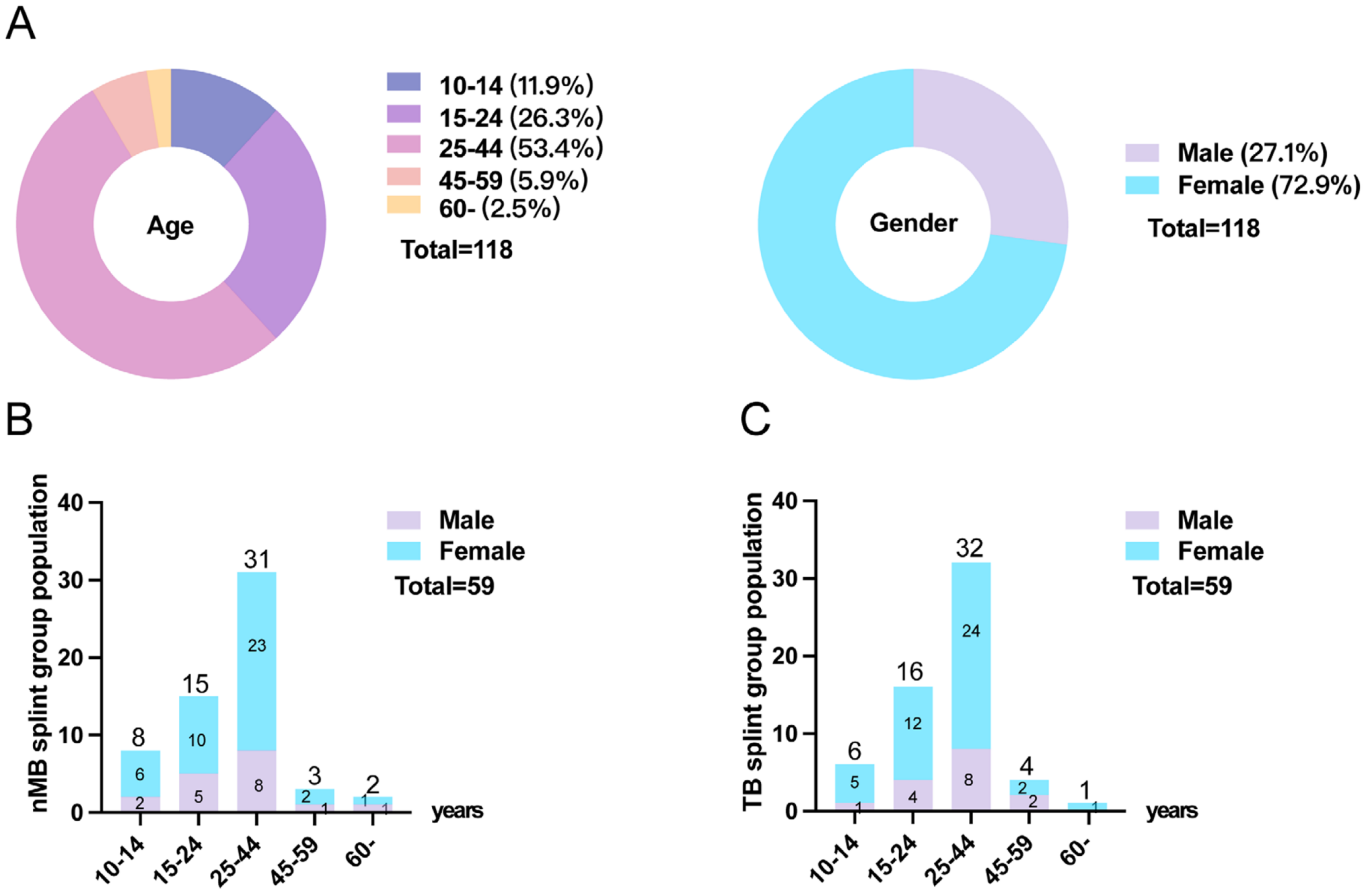
Demographic characteristics of participants and group allocation. (A) Age and gender distribution of all participants (*n* = 118). (B) Age and gender distribution of patients treated with the novel Mono‐Block (nMB) splint (*n* = 59). (C) Age and gender distribution of patients treated with the Twin‐Block (TB) splint (*n* = 59).

A total of 118 patients diagnosed with ADDwR were enrolled in this study. Among them were 32 males (27.1%) and 86 females (72.9%) (Figure 3A). Participants ranged in age from 12 to 68 years. Based on the World Health Organisation's age classification [27, 28, 29], the largest proportion belonged to the early adulthood group (25–44 years), comprising 63 patients (53.4%), while the late adulthood group (≥ 60 years) included only 3 patients (2.5%). Following randomisation, 59 patients were assigned to the nMB splint group and 59 to the TB splint group. The nMB treatment group consisted of 17 males and 42 females (Figure 3B). The TB treatment group consisted of 15 males and 44 females (Figure 3C). There was no significant difference in gender distribution between the two groups (*p* = 0.679). Regarding age distribution, the nMB group comprised 8 adolescents, 15 youths, 31 individuals in early adulthood, 3 in middle adulthood and 2 in late adulthood. The TB group included 6 adolescents, 16 youths, 32 individuals in early adulthood, 4 in middle adulthood and 1 in late adulthood. No statistically significant difference was found in age distribution between the groups (*p* = 0.937).

### 3.3 Efficacy of Joint Noise Reduction With nMB and TB Splint Treatments

**FIGURE 4:**
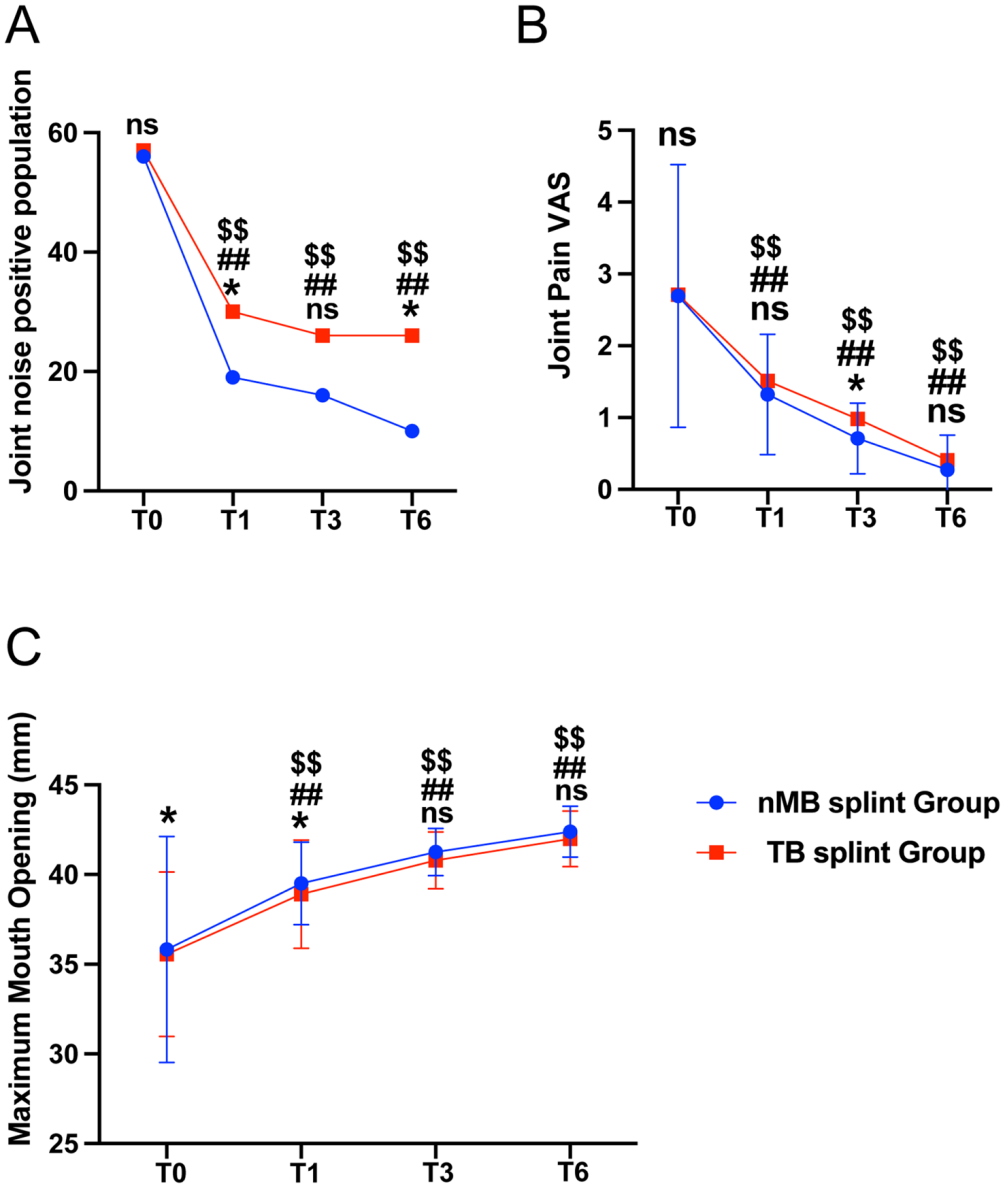
Comparative efficacy of the novel Mono‐Block (nMB) splint and the Twin‐Block (TB) splint in the treatment of anterior disc displacement with reduction (ADDwR). (A) Number of patients with joint noise at each time point in the nMB and TB groups. (B) Visual Analog Scale (VAS) scores for joint pain in both groups at each time point. (C) Maximum mouth opening (MMO) values in both groups at 1 month (T1), 3 months (T3) and 6 months (T6) after treatment. *p < 0.05, nMB group compared with TB group; ^##^ *p* < 0.001, compared to baseline within the nMB group; ^$$^ *p* < 0.001, compared to baseline within the TB group; ns, not significant.

At baseline (T0), joint noise was reported in 56 patients (94.9%) in the nMB splint group and 57 patients (96.6%) in the TB splint group, with no significant difference between groups (*p* = 1.000). Following treatment, the number of patients with joint noise in the nMB group decreased to 19 at 1 month (T1), 16 at 3 months (T3) and 10 at 6 months (T6), corresponding to improvement rates of 66.1%, 71.4% and 82.1%, respectively (all *p* < 0.001 vs. baseline). In the TB group, joint noise was reduced to 30 at T1, 26 at T3 and remained at 26 at T6, with respective efficacy rates of 47.4%, 54.4% and 54.4% (all *p* < 0.001 vs. baseline). Inter‐group comparisons showed significantly greater reductions in joint noise in the nMB group at T1 (*p* = 0.040) and T6 (*p* = 0.001). At T3, the difference approached significance but did not reach statistical significance (*p* = 0.055) (Figure 4A).

### 3.4 Efficacy of Joint Pain Relief With nMB and TB Splint Treatments

Treatment efficacy in alleviating TMD‐related joint pain was assessed using VAS scores. In the nMB splint‐treated group, VAS scores significantly decreased from 2.69 ± 1.83 at T0 to 1.32 ± 0.84 at T1, 0.71 ± 0.49 at T3 and 0.27 ± 0.49 at T6 (all *p* < 0.001). Similarly, the TB splint treatment showed significant pain reduction, with VAS scores declining from 2.71 ± 1.94 at T0 to 1.51 ± 0.92 at 1 month, 0.98 ± 0.57 at 3 months and 0.41 ± 0.56 at 6 months (all *p* < 0.001). Between‐group comparisons revealed no significant differences at T0 (*p* = 0.884), T1 (*p* = 0.195) or T6 (*p* = 0.161). However, at T3, the nMB splint group exhibited significantly greater pain reduction compared to the TB group (*p* = 0.009) (Figure 4B).

### 3.5 Efficacy in Improving Limited Mouth Opening With nMB and TB Splint Treatments

Both the nMB and TB splints significantly improved MMO in patients with ADDwR over the course of treatment. In the nMB group, MMO increased from 35.83 ± 6.31 mm at baseline to 39.51 ± 2.30 mm at T1, 41.26 ± 1.32 mm at T3 and 42.38 ± 1.42 mm at 6 months T6 (all *p* < 0.001). Similarly, in the TB group, MMO improved from 35.56 ± 4.60 mm at T0 to 38.91 ± 3.02 mm at T1, 40.79 ± 1.58 mm at T3 and 41.99 ± 1.55 mm at T6 (all *p* < 0.001). Between‐group comparisons showed significant differences at T0 (*p* = 0.022) and T1 (*p* = 0.035), with the nMB group demonstrating slightly better early outcomes. However, no significant differences were observed at T3 (*p* = 0.083) or T6 (*p* = 0.156) (Figure 4C).

### 3.6 CBCT Imaging Analysis of the Condyle–Fossa Relationship

The condyle‐fossa relationship was directly evaluated by measuring ln(P/A) values at baseline and 6 months post‐treatment in both the nMB and TB splint. In the nMB group, ln(P/A) increased slightly from −0.45 ± 0.31 to −0.38 ± 0.29 (*p* = 0.737), while in the TB group, it changed from −0.54 ± 0.51 to −0.56 ± 0.22 (*p* = 0.601), indicating no significant intra‐group differences. Similarly, inter‐group comparisons at both time points showed no statistically significant differences (T0: *p* = 0.300; T6: *p* = 0.231) (Table 1). Patients with ln(P/A) values < −0.25 were classified as positive for condyle posterior position [10]. We further calculated the positive rate of condyle posterior position as the number of positive joints divided by the total number of joints. In the nMB group, the positive rate decreased from 75% at T0 to 56% at T6 (*p* = 0.264), whereas no change was observed in the TB group (*p* = 1.000). Inter‐group comparisons of positive rates at T0 and T6 also revealed no significant differences (T0: *p* = 1.000; T6: *p* = 0.180) (Table 2).

**TABLE 1:** CBCT images analyse of ln(P/A) at different time points.

	nMB T0 vs. TB T0	nMB T6 vs. TB T6	nMB T0 vs. nMB T6	TB T0 vs. TB T6
ln(P/A)	−0.45 ± 0.31 (−0.54 ± 0.51)	−0.38 ± 0.29 (−0.56 ± 0.22)	−0.45 ± 0.31 (−0.38 ± 0.29)	−0.54 ± 0.51 (−0.56 ± 0.22)
*p*	0.300	0.231	0.737	0.601

*Note:* Pairwise comparisons between groups were conducted using the paired *t*‐test, Wilcoxon signed‐rank test, Mann–Whitney *U* test, and independent samples *t*‐test, as appropriate based on data distribution and pairing. Values are presented as mean ± standard deviation. Percentages outside parentheses refer to the nMB group, while percentages inside parentheses refer to the TB group.

**TABLE 2:** Positive rate of condyle posterior position at different time points.

	nMB T0 vs. TB T0	nMB T6 vs. TB T6	nMB T0 vs. nMB T6	TB T0 vs. TB T6
Positive rate	75% (78%)	56% (78%)	75% (56%)	78% (78%)
*p*	1.000	0.180	0.264	1.000

*Note: χ* ^2^ test was performed to assess differences in positive rates between different time periods. Values are presented as percentage within each group. Values are presented as percentages within each group. Numbers outside parentheses represent the nMB group; numbers inside parentheses represent the TB group.

### 3.7 Satisfaction Ratings

**FIGURE 5:**
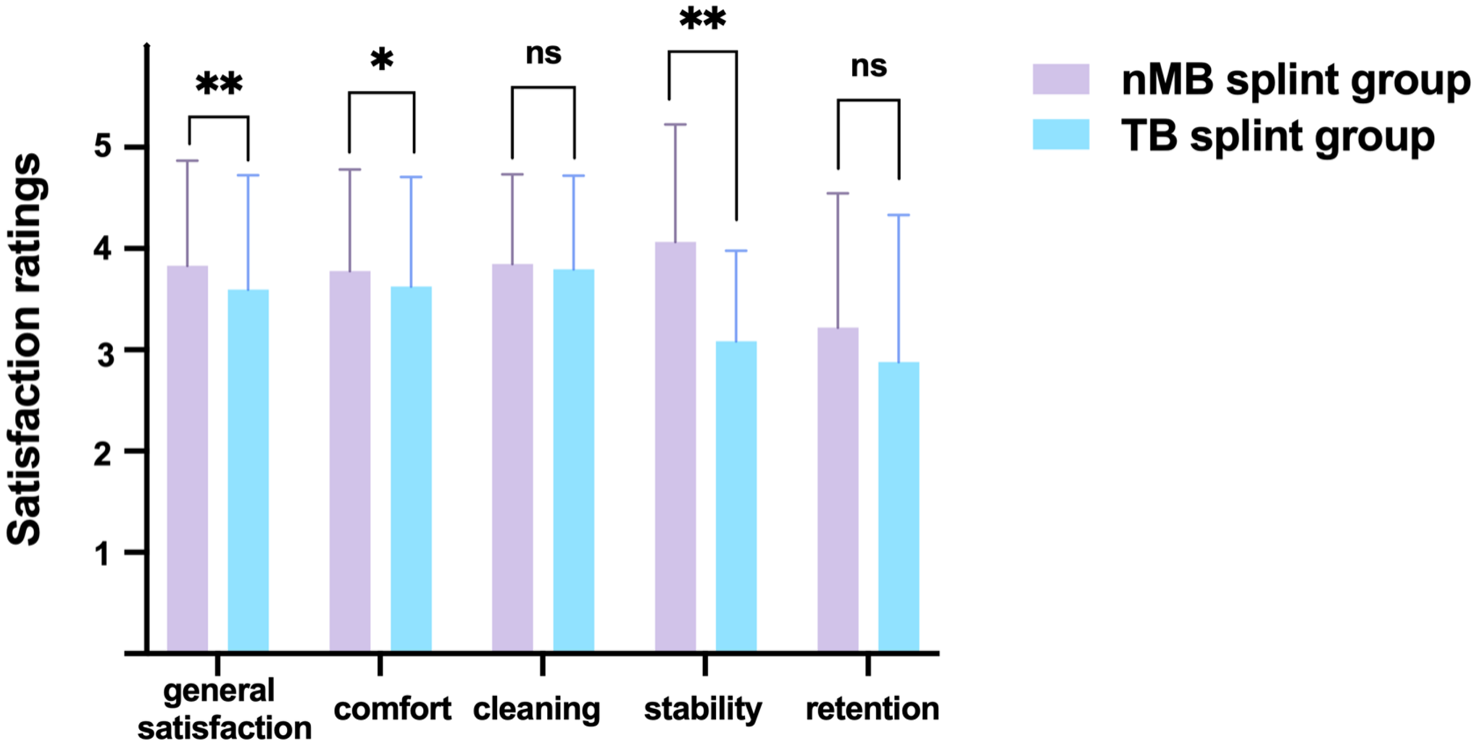
Visual Analog Scale (VAS) assessment of patient satisfaction after 6 months (T6) of treatment. Satisfaction ratings were compared between the novel Mono‐Block (nMB) splint group and the Twin‐Block (TB) splint group across five domains: Overall satisfaction, ease of cleaning, comfort, stability and retention. Data are presented as mean ± SD. *p < 0.05, **p < 0.001, nMB group compared with TB group; ns, not significant.

To compare patient satisfaction between the nMB and TB splint groups, five parameters were assessed using the VAS: general satisfaction, comfort, ease of cleaning, stability and retention. In the nMB group, the mean scores were as follows: general satisfaction 3.83 ± 1.04, comfort 3.78 ± 1.00, cleaning 3.85 ± 0.89, stability 4.07 ± 1.16 and retention 3.22 ± 1.33. In the TB group, the corresponding scores were 3.05 ± 1.02, 3.27 ± 1.03, 3.80 ± 0.92, 3.08 ± 0.87 and 2.88 ± 1.45. The nMB splint group showed significantly higher ratings in general satisfaction (*p* < 0.001), comfort (*p* = 0.005) and stability (*p* < 0.001) compared to the TB group. However, no significant differences were observed in ease of cleaning (*p* = 0.771) or retention (*p* = 0.195) (Figure 5).

## 4 Discussion

ARS has been widely recognised as an effective conservative treatment for TMD [15, 19, 30, 31, 32, 33, 34]. Unlike the conventional TB appliance used in orthodontics, the TB splint applied in our study for ADDwR treatment was specifically modified and did not include components such as arch‐expansion elements. This randomised controlled trial compared the therapeutic outcomes of the nMB splint with the TB splint in managing ADDwR. Our findings indicate that the nMB splint offers superior clinical benefits in joint noise reduction and improved comfort, making it a promising conservative treatment option for ADDwR, despite showing similar outcomes in disc position reduction and pain relief.

ARS therapy is a non‐invasive, reversible intervention widely used in the management of TMD and ADDwR. Its therapeutic mechanism involves guiding the mandible into a minimal protruded position where joint noise is eliminated, thereby promoting relaxation of the masticatory muscles and reducing compressive forces on the posterior aspect of the TMJ. This decompression may enhance local vascular perfusion and facilitate the redistribution of intra‐articular space, creating a biomechanically favourable environment for disc recapture. Consequently, ARS therapy supports the restoration of functional coordination among the condyle, articular disc and joint fossa, thereby achieving therapeutic efficacy in patients with ADDwR [13, 35, 36]. In contrast, the stabilisation splint (SS) maintains the mandible in a neutral or ‘natural’ position, preventing displacement, and has been reported to increase the superior TMJ joint space while reducing joint load and masticatory muscle tension through even occlusal contact [37]. The SS does not directly reposition the articular disc; its primary mechanism for pain relief is to alleviate muscle tension and decrease abnormal joint stress. The nMB splint developed in this study retains the core therapeutic functions of conventional ARS appliances while incorporating structural refinements designed to enhance clinical efficacy and improve patient comfort.

Joint noise is a common symptom in patients with ADDwR [38, 39], and may be associated with abnormalities in joint fluid dynamics, disc‐condyle coordination or condylar morphology [40, 41]. The reduction of joint noise is an important clinical indicator of symptom relief. ARS has demonstrated clinical efficacy in alleviating joint noise symptoms [16, 42, 43, 44]. In this study, the nMB group exhibited significantly greater improvement in joint noise relief compared to the TB group at both the 1‐month (T1: 66.1% vs. 47.4%) and 6‐month (T6: 82.1% vs. 54.4%) follow‐ups, although no significant difference was observed at the 3‐month (T3) follow‐up. Importantly, the overall therapeutic efficacy was superior to that of the TB splint. This benefit is likely attributable to the distinctive design features of the nMB splint, which allow for precise and stable mandibular advancement with a retention system that effectively maintains the therapeutic position. This design is particularly beneficial during sleep, when minimising mandibular positional drift is critical for sustaining disc‐condyle coordination. In contrast, the TB splint relies on the occlusal inclines of separate maxillary and mandibular components. Such a mechanism may be more susceptible to displacement during nocturnal use, especially in patients with mouth breathing or muscular relaxation, thereby compromising the consistency of disc recapture [18, 19, 20]. The sustained clinical benefit of the nMB splint in alleviating joint noise underscores its potential to more effectively reestablish a stable and functional disc‐condyle relationship within our study period. However, the disappearance of joint noise may also indicate that ADDwR has progressed to anterior disc displacement without reduction (ADDwoR).

ARS also contributed to patients' pain alleviation [45, 46] by unloading TMJ structures, including retrodiscal tissue and masticatory muscles [47, 48, 49]. VAS scores indicated significant reductions in joint pain for both groups following treatment (nMB splint group: 2.69 ± 1.83 to 0.27 ± 0.49; TB splint group: 2.71 ± 1.94 to 0.41 ± 0.56). Notably, the nMB splint group showed lower VAS scores compared with the TB splint group at 3 months (T3), whereas no significant differences were observed between the groups at 1 month (T1) or 6 months (T6). Previous studies have indicated that occlusal splints may alleviate pain through muscle relaxation [50, 51, 52]; however, the precise mechanisms underlying these effects have not yet been fully elucidated [53]. In our study, both splints demonstrated effectiveness in alleviating pain within 6 months of treatment. However, no direct assessment of muscle activity was performed. In future studies, we will utilise electromyography to analyse changes in muscle activity and identify the mechanisms underlying muscle pain relief.

Both groups exhibited significant increases in MMO following treatment (nMB splint group: 35.83 ± 6.31 mm to 42.38 ± 1.42 mm; TB splint group: 35.56 ± 4.60 mm to 41.99 ± 1.55 mm). Throughout the treatment period, both splint designs demonstrated comparable efficacy in achieving favourable therapeutic outcomes. This suggests that both splints contribute to the relaxation of periarticular musculature and promote mandibular advancement, thereby increasing the intra‐articular space. As a result, the condyles may seat more centrally in the glenoid fossa, eliminating mechanical interference from the displaced disc during condylar translation, which ultimately facilitates the restoration of normal mandibular opening [14, 54].

Previous studies have proposed that the mechanism of ARS involves ‘recapturing’ of the disc by downward and forward movement of the condyle [14]. However, no significant changes were observed in the positive rate of condyle posterior position following treatment with the nMB splint and the TB splint. As noted by Chen, the stability of the disc‐condyle relationship often cannot be maintained upon ARS removal in the majority of patients, which may explain the limited improvement in condyle posterior position rates in the present study [55]. In conservative therapy, the primary objective is to alleviate patient symptoms, including reducing pain, diminishing joint noise and improving mandibular function by increasing MMO. Furthermore, disc position cannot be accurately evaluated based solely on CBCT joint space; a reliable assessment requires both MRI and a comprehensive clinical examination [10].

While demonstrating therapeutic efficacy comparable to the TB splint, the nMB splint showed superior outcomes in terms of general satisfaction, comfort and stability. This result aligns with our original intent behind this novel splint, which aimed to optimise patient compliance by improving usability. We sought to optimise patient compliance to extend splint wear time, as previous studies have suggested that prolonged use of occlusal appliances may contribute to improved long‐term therapeutic outcomes [56, 57].

This study has several important limitations that should be taken into account when interpreting the findings. MRI scanners are not yet widely available in the dental field, and their high cost further limits their routine use. Therefore, in daily clinical practice, CBCT is generally employed as an auxiliary imaging modality for assessing the dental arch, osseous structures and joint space. However, CBCT cannot directly indicate changes in the muscle and joint disc. Even the DC/TMD acknowledges the low sensitivity of clinical assessment for objectively diagnosing anterior disc displacement. In the present study, the morphology and position of the articular disc remain unknown. MRI remains the gold standard for imaging soft tissues of the TMJ, and future studies incorporating MRI would provide more definitive evidence of treatment efficacy [10]. The open‐label design of this trial also represents a limitation. Due to the apparent differences between the two splint designs, blinding of patients and treating clinicians was not feasible. Clinical assessments of joint noise and MMO were performed without blinding. However, CBCT imaging analysis and statistical analyses were conducted by investigators who were blinded to group allocation. Although the statistician remained blinded during data analysis, the lack of blinding in other aspects may have introduced observer or performance bias. Another limitation concerns the study setting. The trial was conducted at a single‐centre with a relatively homogeneous patient population, which may restrict the generalizability of the findings to broader populations and clinical contexts. Furthermore, the follow‐up period was limited to 6 months. Temporomandibular disorders are chronic conditions, and a longer follow‐up is necessary to determine the durability of the observed therapeutic effects and to identify potential relapse. The absence of a placebo or conservative management control group also limits the interpretation of the present findings. Without comparison to standard conservative care (such as education, counselling or exercise therapy), it is not possible to fully distinguish the specific therapeutic effects of splint therapy from the natural course of the condition or nonspecific placebo effects. Moreover, no orthodontic follow‐up was performed. Prolonged mandibular advancement with anterior repositioning splints may result in occlusal alterations, unwanted tooth movement, or other dental side effects, which could not be assessed in this trial. Future studies with larger samples, longer follow‐up, orthodontic monitoring and inclusion of appropriate control groups are warranted to confirm both the safety and therapeutic effectiveness of splint therapy.

Our study demonstrates that the nMB splint, as a novel ARS design, provides comparable improvements in mouth opening and joint pain relief to the TB splint, while offering superior reduction in joint noise and enhanced patient comfort. These advantages are likely attributed to its stable, integrated structure. Overall, the nMB splint represents a promising innovation of ARS therapy and warrants further evaluation in larger‐scale and imaging‐supported clinical studies.

## 5 Conclusion

The nMB splint demonstrated superior efficacy over the TB splint in reducing joint noise and enhancing patient comfort, with higher patient‐reported satisfaction in terms of comfort, retention and stability. Both splints showed comparable effectiveness in improving MMO, relieving joint pain. These findings suggest that the nMB splint is a promising, patient‐oriented advancement in ARS therapy for the conservative management of ADDwR.

## Author Contributions

**Zhilei Liu:** data collection, writing – original draft, formal analysis, validation, visualisation. **Antong Wu:** data collection, writing – original draft, funding acquisition, conceptualization, methodology, writing – review and editing. **Wenyi Cai:** data collection, writing – review and editing. **Jiaqian Fan:** validation, methodology, writing – review and editing. **Yunyi Yuan:** writing – review and editing, data collection, methodology. **Yufu Lin:** data collection, methodology. **Rong Zhang:** project administration, data collection, methodology. **Qingbin Zhang:** project administration, supervision, writing – review and editing. **Wei Cao:** funding acquisition, conceptualization, supervision, project administration, supervision, writing – review and editing.

## Funding

This research was funded by the National Key Research and Development Program of China (2023YFC2509200), the Research Capability Enhancement Program of Guangzhou Medical University (No. 2024SRP162), and the Medical Science and Technology Research Foundation of Guangdong Province (No. A2024532).

## Ethics Statement

The study was conducted in accordance with the Declaration of Helsinki and was approved by the Ethics Committee of the Affiliated Stomatology Hospital of Guangzhou Medical University (Approval number: LCYJ20250618004).

## Consent

The authors have nothing to report.

## Conflicts of Interest

The authors declare no conflicts of interest.

## Supporting information


**Figure S1:** Representative images of the novel Mono‐Block (nMB) splint and the Twin‐Block (TB) splint. (A, B) Lateral and occlusal views of the nMB splint. (C, D) Lateral and occlusal views of the TB splint.
**Figure S2:** Schematic comparisons of the novel Mono‐Block (nMB) splint and the Twin‐Block (TB) splint. (A–C) Schematic comparisons of the nMB splint. (D–F) Schematic comparisons of the TB splint.

## Data Availability

The corresponding author, D.D.S. Dr. W. Cao, had full access to the data in the study and takes responsibility for the integrity of the data and the accuracy of the data analysis. The data and materials that support the findings of this study are available from the corresponding author upon reasonable request.
