# Supplementary material for: Effectiveness of a Novel Mono‐Block Splint Versus a Twin‐Block Splint for Anterior Disc Displacement With Reduction: A Randomised Controlled Trial
Source: J Oral Rehabil. 2026 Mar 7;53(7):1275–85. doi: 10.1111/joor.70179 (PMC13261787; doi:10.1111/joor.70179)
Supplement: Supplementary file 1 — Figure S1: Representative images of the novel Mono‐Block (nMB) splint and the Twin‐Block (TB) splint. (A, B) Lateral and occlusal views of the nMB splint. (C, D) Lateral and occlusal views of the TB splint. Figure S2: Schematic comparisons of the novel Mono‐Block (nMB) splint and the Twin‐Block (TB) splint. (A–C) Schematic comparisons of the nMB splint. (D–F) Schematic comparisons of the TB splint. [file JOOR-53-1275-s001.docx]

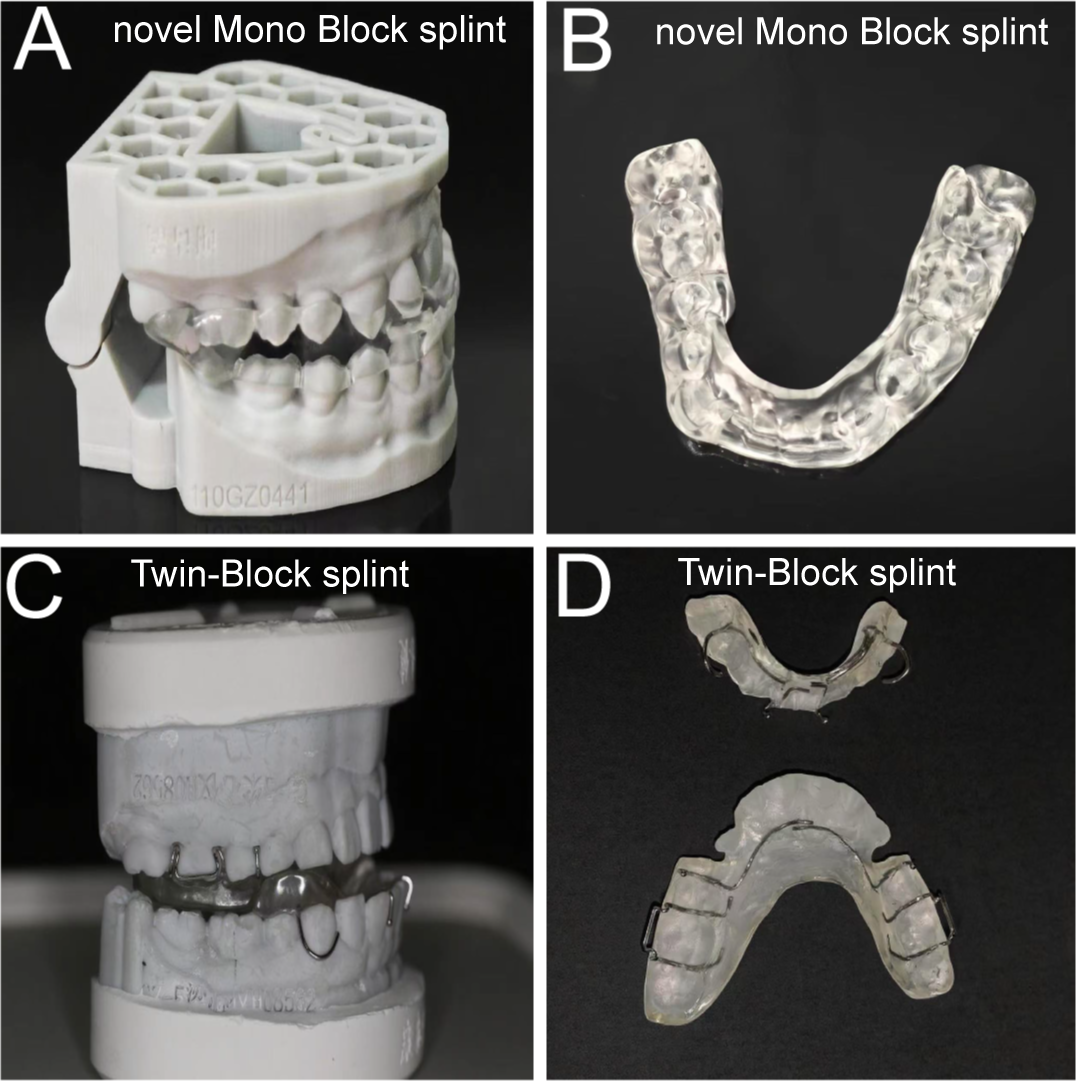


**Supplementary Figure 1.** **Representative images of the novel Mono-Block (nMB) splint and the Twin-Block (TB) splint.** (A–B) Lateral and occlusal views of the nMB splint. (C–D) Lateral and occlusal views of the TB splint.


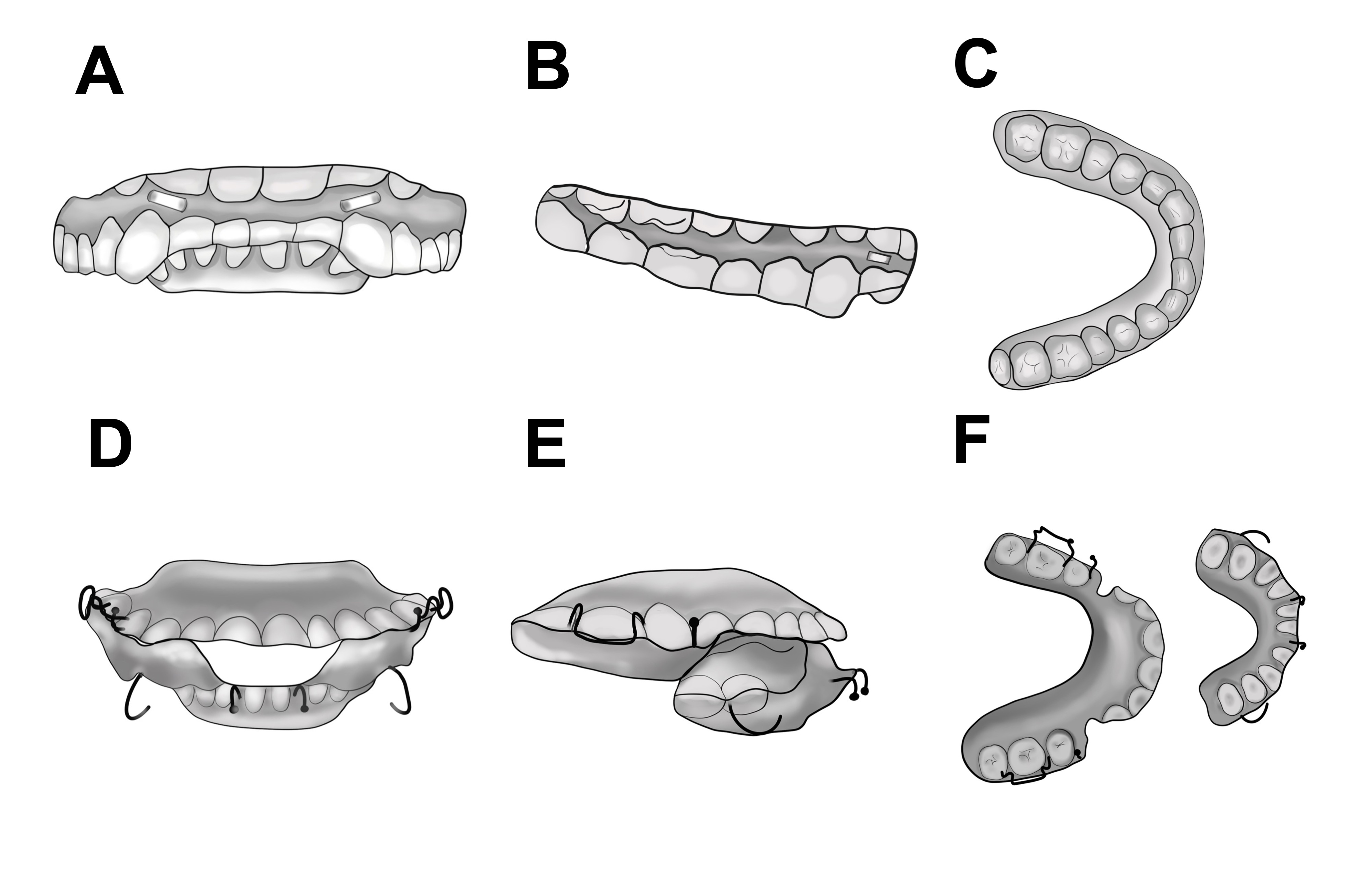


**Supplementary Figure 2. Schematic comparisons of the novel Mono-Block (nMB) splint and the Twin-Block (TB) splint.** (A–C) Schematic comparisons of the nMB splint. (D–F) Schematic comparisons of the TB splint.
